# Development of energy demand and carbon emission dataset for Nile University of Nigeria

**DOI:** 10.1016/j.dib.2023.109347

**Published:** 2023-06-29

**Authors:** Tahir A. Zarma, Paul O. Micheal, Ahmadu A. Galadima, Tologon Karataev, Adekunle Adeleke, Oghenewvogaga Oghorada, Hussein U. Suleiman

**Affiliations:** a Nile University of Nigeria, Abuja. Address: Plot 681, Cadastral Zone C-OO, Research & Institution Area Nigeria, Airport Rd, Jabi 900001, Abuja, Nigeria; b National Space Research and Development Agency, Abuja. Address: Obasanjo Space Centre, Umaru Musa Yar'adua Express Way, P.M.B. 437, Garki, Abuja, Nigeria

**Keywords:** Energy demand, Load demand, Carbon emission, Sizing of renewable energy sources

## Abstract

The global energy crisis and ozone layer depletion as a result of carbon emissions have increased the awareness and acceptance of renewable energy sources as an alternative form of electric power, resulting in the sizing of renewable energy sources. However, in order to properly size an energy power system, the information being addressed, such as the load demand, is critical. The Load demand data of Nile University campus is obtained from one of its power stations (PS-1) for a period of eight month. The data was measured from the bus bar of the power station using smart meters on a weekly basis. To power the university campus, the diesel generators are synchronized using Genset controllers with suitable communications interfaces and a SMA hybrid controller, which continually checks the power output of the power sources as well as the working condition of all loads in the busbar. The diesel generators are synchronized using SMA hybrid controllers and combined with the other source of the energy at a common bus bar and used to power the university campus. Additionally, carbon emission data were obtained from the PV solar system reading.

**Specifications Table**

**Table:** 

Subject	Energy
Specific subject area	Energy Load demand is very crucial in sizing electrical power generators. Furthermore, renewable energy sources are used to curb carbon emissions. The need for energy demand data for effective sizing of renewable of energy systems.
Type of data	Table
How the data were acquired	The data set was acquired by measuring the weekly load driven by the generating set in the power – the utility grid, diesel engines, and PV system. The data was measured using current transformers also known smart meters. This meter is connected in series with each generating set and the common bus bar of the power station 1.
Data format	Raw Analyzed
Description of data collection	The PS-1 of Nile University is composed of three diesel generators, the public utility energy grid, and an on-grid PV solar system. First off, the diesel generators are synchronized into a single output and then connected in parallel with the other sources at the PS-1 bus bar. Furthermore, Current transformers (Smart meters) are then used in series with each generating set to measure the weekly load consumption. The weekly data load consumption was however obtained from the previous week as shown in the data.
Data source location	• Institution: Nile University of Nigeria, Abuja • City/Town/Region: Jabi/FCT/Abuja • Country: Nigeria • Latitude and longitude (and GPS coordinates, if possible) for collected samples/data: 9.01341510693328, 7.396425820857888
Data accessibility	Aja Zarma, Tahir; Micheal, Paul; Karataev, Tologon; Adamu Galadima, Ahamadu; Adekunle, Adeleke; Usman Suleiman, Hussein; Oghonuvwembe, Oghorada (2023), “Energy Consumption Dataset”, Mendeley Data, V3, doi:10.17632/d6tzhvp8pr.

## Value of the Data

• The data set can be used as base for determining energy demand of institutions.
• The data be used in sizing of renewable energy system with similar load demand.
• The data can be used to obtain the carbon emission savings.
• The data can be used for load forecasting and power system planning.
• The data can be used by industry and academic institution for research development.
• The data can be used in optimization and machine learning Algorithms.

## 1 Objective

The objective behind the development of this data is because carbon emissions are the major contributors towards global warming. However, this can be curbed with increase in penetration of renewable energy sources. Accordingly, the renewable energy source can only be implemented if the load being addressed is known. Therefore, these data were developed to determine the load demand and carbon emission of Nile University of Nigeria.

## 2 Data Description

In this section, the energy demand data measured are discussed in detail. The three sources of power from which the data was measured are the public utility grid, the Photovoltaic Energy system, and the synchronized diesel engines. Furthermore, carbon emission savings data obtained from the use of PV solar system is also presented.

## 3 Energy Demand Data

This paper presents electricity energy demand dataset of Nile University of Nigeria, Abuja. It consists of data obtained from three different source of electrical energy generation systems. As shown in the table, the dataset corresponds to the load driven by these generating systems. However, the data obtained was on weekly basis for a period spanning over a period of 30 weeks.

**Fig. 1:**
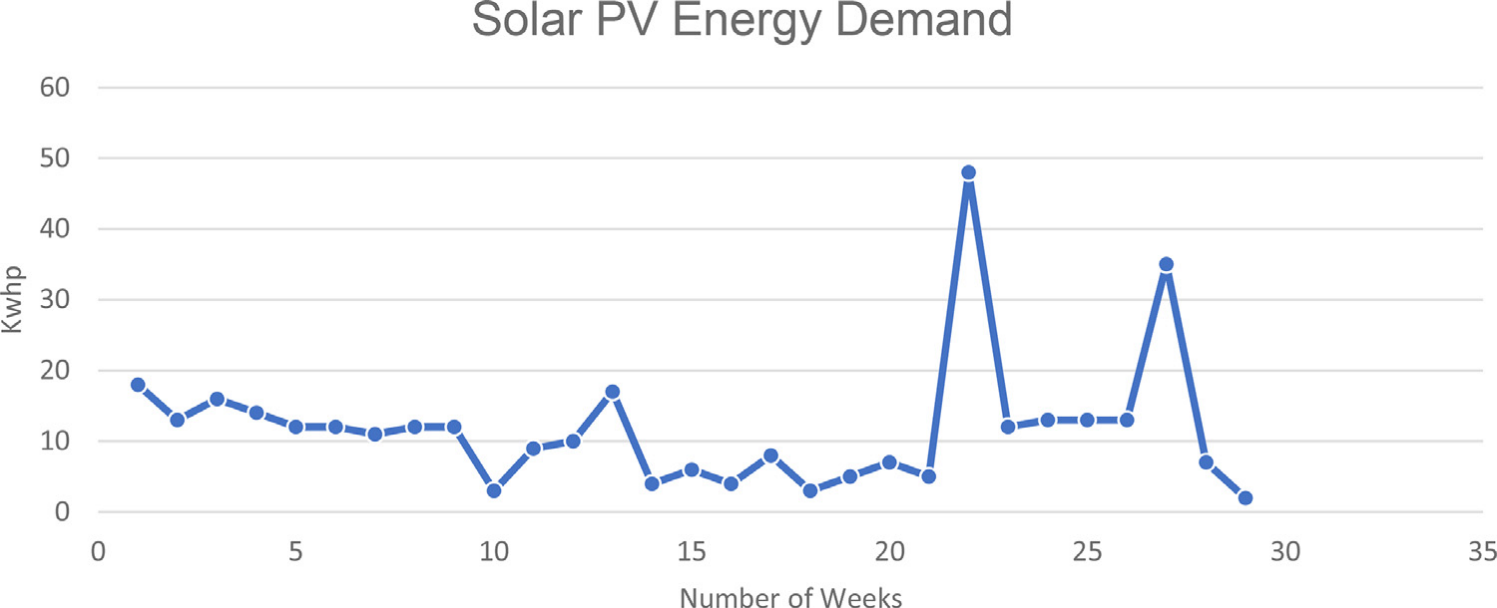
PV Solar energy demand. The data obtained for the solar PV system for the year round is tabulated in Table 1

Additionally, the power station is characterized by a grid connected PV solar system, a public utility energy grid and three synchronized diesel engines. Fig. 1. shows the weekly energy in Kw-h demand serviced by the PV solar system of Nile University for Period of 30 weeks. Furthermore, the SMA meters and SMA controller were available for the period of 30 weeks only and as such the data that was captured was for only the mentioned period. However, with access to the SMA server, were able to gather the year-round solar PV yield and is presented in Table 1.

**Table 1:** Monthly Solar PV Yeild.

Time period	Total yield [MWh]	Average yield expectations [MWh]
Jan-22	51.143	105.053
Feb-22	51.953	90.437
Mar-22	64.002	85.869
Apr-22	55.308	69.426
May-22	57.196	60.291
Jun-22	51.915	52.07
Jul-22	37.683	52.07
Aug-22	27.739	55.724
Sep-22	23.967	65.772
Oct-22	45.066	79.475
Nov-22	55.365	93.177
Dec-22	47.542	104.139

The data in Table 2 shows the weekly measurements of the pv solar energy consumption.

### 3.1 Solar PV energy data

The data of Table 2 shows the weekly energy load addressed with the PV solar system. The average Kilowatts-hour service by the solar system is approximately 344 kWh. The weekly distribution is as shown in Fig. 1.

**Table 2:** PV Energy Consumption.

S/N	MONTH	WEEK	PREVIOUS READING	CURRENT READINGS	SOLAR ENERGY (KWh)
1	Month 1	week 1(may 6−13)	362	380	18
2		week 2(may 13–20)	380	393	13
3		week 3(may 20–27)	393	409	16
4		4(may 27–3rd june)	278	292	14

5	Month 2	week 5(june 3rd-10th june	292	304	12
6		week 6(june 10th-june 17th)	304	316	12
7		week 7(june 17th-june 24th)	316	327	11
8		week 8(june 24th-july 1st)	327	339	12

9	Month 3	week 9(july 1st-july 8th)	339	351	12
10		week 10(july 8th-july 15th)	351	354	3
11		week 11(july 15th-july 22nd)	354	363	9
12		week 12(july 22nd-july 29th)	363	373	10

13	Month 4	Week 13–14 (July 29th-Aug 12th)	373	390	17
14		Aug 12th-19th	390	394	4
15		Aug 19th-26th	394	400	6
16		Aug 26th-Sept 2nd	400	404	4

17	Month 5	Sept 2nd-9th	404	412	8
18		Sept 9th-16th	412	415	3
19		Sept 16th-23rd	415	420	5
20		Sept 23rd- 30th	420	427	7

21	Month 6	Sept 30th-Oct 7th	427	432	5
22		Oct 7th-Nov 4th	432	480	48
23		Nov 4th-11th	480	492	12
24		Nov 11th-18th	492	505	13

25	Month 7	Nov 18th-25th	505	518	13
26		Nov 25th- Dec 2nd	518	531	13
27		Dec 2nd-23	531	566	35

28	Month 8	Dec 23rd-30th	566	573	7
29		Dec 30th-Jan 6th	573	575	2

**Total**					**344**

### 3.2 Carbon emission savings data

The Solar PV system been a renewable energy system, has zero carbon emission as such the usage of the Solar energy has given rise carbon emission savings. The emission of carbon gasses has adverse effect on the planet this is because of the ozone layer and could give rise to global warming. The carbon emission saving data of Table 3 were obtained using Eq. (1).

(1) $$Co_{2}\;Savings=Kwh\times Co_{2}\;factor$$

**Table 3:** Carbon emission savings.

S/N	MONTH	WEEK	PREVIOUS READING	CURRENT READINGS	SOLAR ENERGY (KWh)	Weekly Carbon Emission Savings	Cummulative Carbon Emission
1	Month 1	week 1(may 6−13)	362	380	18	**10.62**	**10.62**
2		week 2(may 13–20)	380	393	13	**7.67**	**18.29**
3		week 3(may 20–27)	393	409	16	**9.44**	**27.73**
4		4(may 27–3rd june)	278	292	14	**8.26**	**35.99**

5	Month 2	week 5(june 3rd-10th june	292	304	12	**7.08**	**43.07**
6		week 6(june 10th-june 17th)	304	316	12	**7.08**	**50.15**
7		week 7(june 17th-june 24th)	316	327	11	**6.49**	**56.64**
8		week 8(june 24th -july 1st)	327	339	12	**7.08**	**63.72**

9	Month 3	week 9(july 1st-july 8th)	339	351	12	**7.08**	**70.80**
10		week 10(july 8th-july 15th)	351	354	3	**1.77**	**72.57**
11		week 11(july 15th-july 22nd)	354	363	9	**5.31**	**77.88**
12		week 12(july 22nd-july 29th)	363	373	10	**5.90**	**83.78**

13	Month 4	Week 13–14(July 29th-Aug 12th)	373	390	17	**10.03**	**93.81**
14		Aug 12th-19th	390	394	4	**2.36**	**96.17**
15		Aug 19th-26th	394	400	6	**3.54**	**99.71**
16		Aug 26th-Sept 2nd	400	404	4	**2.36**	**102.07**

17	Month 5	Sept 2nd-9th	404	412	8	**4.72**	**106.79**
18		Sept 9th-16th	412	415	3	**1.77**	**108.56**
19		Sept 16th-23rd	415	420	5	**2.95**	**111.51**
20		Sept 23rd- 30th	420	427	7	**4.13**	**115.64**

21	Month 6	Sept 30th-Oct 7th	427	432	5	**2.95**	**118.59**
22		Oct 7th-Nov 4th	432	480	48	**28.32**	**146.91**
23		Nov 4th-11th	480	492	12	**7.08**	**153.99**
24		Nov 11th-18th	492	505	13	**7.67**	**161.66**

25	Month 7	Nov 18th-25th	505	518	13	**7.67**	**169.33**
26		Nov 25th- Dec 2nd	518	531	13	**7.67**	**177.00**
27		Dec 2nd-23	531	566	35	**20.65**	**197.65**

28	Month 8	Dec 23rd-30th	566	573	7	**4.13**	**201.78**
29		Dec 30th-Jan 6th	573	575	2	**1.18**	**202.96**

**Total**					**344**	**Total**	**202.96**

**Fig. 2:**
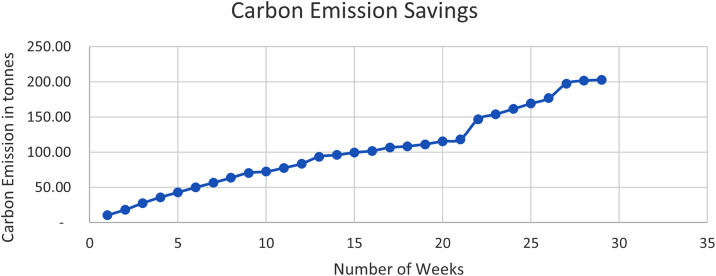
Carbon emission savings.

Where the carbon emission factor is unique for every country in any part of the world. However, the factor considered for Nigeria is 0.59 [1]. The carbon emission savings are as shown in Table 3. Furthermore, Fig. 2 shows the weekly plot of the emission savings with total of **202.96 tons** of gasses saved.

### 3.3 Diesel generators data

**Fig. 3:**
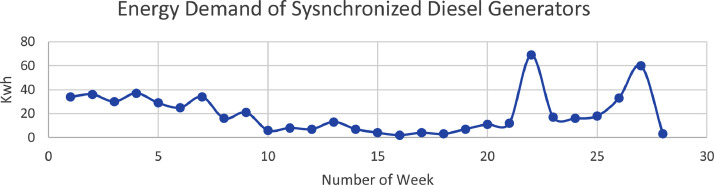
Diesel engine energy demand.

As mentioned earlier, the four synhcronised diesel engines have a combined capacity of 4.3MW. The data of Table 4 shows the weekly energy consumed by the load which is addressed by the diesel generators. There are four diesel generators synchronized in parallel. Fig. 3 shows the energy demand distribution over a period of 30 weeks.

**Table 4:** Energy Consumption from Diesel Generators.

			GENERATOR CONTRIBUTION(MWH)		
S/N	Month	week	previous reading	current reading	Generator (MWh)
1	Month 1	week 1(may 6−13)	622	656	**34**
2		week 2(may 13–20)	656	692	**36**
3		week 3(may 20–27)	692	722	**30**
4		4(may 27–3rd june)	421	458	**37**

5	Month 2	week 5(june 3rd-10th june	458	487	**29**
6		week 6(june 10th -june 17th)	487	512	**25**
7		week 7(june 17th -june 24th)	512	546	**34**
8		week 8 (june 24th -july 1st)	546	562	**16**

9	Month 3	week 9(july 1st-july 8th)	562	583	**21**
10		week 10(july 8th -july 15th)	583	589	**6**
11		week 11 (july 15th-july 22nd)	589	597	**8**
12		week 12 (july 22nd-july 29th)	597	604	**7**

13	Month 4	Week 13–14 (July 29th-Aug 12th)	604	617	**13**
14		Aug 12th-19th	617	624	**7**
15		Aug 19th-26th	624	628	**4**
16		Aug 26th-Sept 2nd	628	630	**2**

17	Month 5	Sept 2nd-9th	630	634	**4**
18		Sept 9th-16th	634	637	**3**
19		Sept 16th-23rd	637	644	**7**
20		Sept 23rd- 30th	644	655	**11**

21	Month 6	Sept 30th-Oct 7th	655	667	**12**
22		Oct 7th-Nov 4th	667	736	**69**
23		Nov 4th-11th	736	753	**17**
24		Nov 11th-18th	753	769	**16**

25	Month 7	Nov 18th-25th	769	787	**18**
26		Nov 25th-Dec 2nd	787	820	**33**
27		Dec 2nd-23	820	880	**60**

28	Month 8	Dec 23rd-30th	880	883	**3**
29		Dec 30th-Jan 6th	883	913	10

Total					**572**

### 3.4 Effects of running generators

The synchronized generators have a combined energy contribution of 572MW for the period of data collected. This has a significant effect on the environment because of the carbon emissions due to combustion of diesel. Consequently, the carbon emission as results of using the generators are as obtained using Eq. (2).

(2) $$E=\frac{Q\times E_{c}\times E_{F}}{1000}$$

where E is the total emissions released measured in tones CO_2-e,_ Q is the quantity of fuel combusted in kL, E_C_ is energy content factor of the fuel in GJ/kL E_F_ is the emission factor for the fuel in Kg CO2. Furthermore, the estimate of the energy consumption for the eight month is as Table 5.

**Table 5:** Energy Consumption of Diesel Generators.

S/N	Generator Capacity			Generators Operation time		fuel Consumption	Fuel Use	Generated Power
1	Load%	kW	Consumption L/h	Daily Hours	Eight months Hours	L/kWh	kL	MWh
2	100	2600	256	6	1458	0.259	4040.4	15,600
3	75	1950	194	6	1458	0.194	2269.8	11,700
3	50	1300	130	8	1944	0.13	1352	10.400
4	25	650	65	4	972	0.065	169	2600

		Total Q					7831.2	

Using an E_C_ of 38.6 GJ/kL and an E_F_ of 69.5Kg CO2 -e per GJ the greenhouse gas emission is obtained as 21,008.22 tones.

### 3.5 The public utility grid

**Fig. 4:**
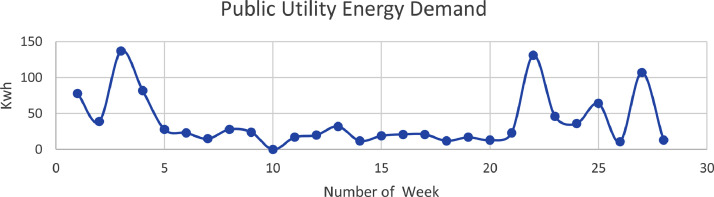
public utility energy demand.

The third source of electric energy is the public utility energy grid (Table 6). Which is the Abuja Electricity Distribution company. This is the highest used source of energy in terms load demand. The energy consumption data of the load demand as shown in Table 4. This is the highest with almost 1000 kwh. The distribution over a period of 30 weeks is as shown in Fig. 4.

**Table 6:** Energy Data Obtained from the Public Utility Grid.

S/N	Month	week	previous reading	current reading	Grid (MWh)
			**940**	**1018**	**78**
1	Month 1	week 1(may 6 −13)	**1018**	**1057**	**39**
2		week 2(may 13–20)	**1057**	**1194**	**137**
3		week 3(may20–27)	**671**	**753**	**82**
4		4(may 27–3rd june)	**753**	**781**	**28**

5	Month 2	week 5(june 3rd-10th june	**781**	**804**	**23**
6		week 6(june 10th -june 17th)	**804**	**819**	**15**
7		week 7(june 17th -june 24th)	**819**	**847**	**28**
8		week 8 (june 24th -july 1st)	**847**	**871**	**24**

9	Month 3	week 9(july 1st-july 8th)	**871**	**871**	**0**
10		week 10(july 8th -july 15th)	**871**	**888**	**17**
11		week 11 (july 15th-july 22nd)	**888**	**908**	**20**
12		week 12 (july 22nd-july 29th)	**908**	**940**	**32**

13	Month 4	Week 13–14 (July 29th-Aug 12th)	**940**	**952**	**12**
14		Aug 12th-19th	**952**	**971**	**19**
15		Aug 19th-26th	**971**	**992**	**21**
16		Aug 26th-Sept 2nd	**992**	**1013**	**21**

17	Month 5	Sept 2nd-9th	**1013**	**1025**	**12**
18		Sept 9th-16th	**1025**	**1042**	**17**
19		Sept 16th-23rd	**1042**	**1055**	**13**
20		Sept 23rd- 30th	**1055**	**1078**	**23**

21	Month 6	Sept 30th-Oct 7th	**1078**	**1209**	**131**
22		Oct 7th-Nov 4th	**1209**	**1255**	**46**
23		Nov 4th-11th	**1255**	**1291**	**36**
24		Nov 11th-18th	**1291**	**1355**	**64**

25	Month 7	Nov 18th-25th	**1355**	**1366**	**11**
26		Nov 25th- Dec 2nd	**1366**	**1473**	**107**
27		Dec 2nd-23	**1473**	**1486**	**13**

28	Month 8	Dec 23rd-30th	1486	1494	9
29		Dec 30th-Jan 6th	1494	1519	15

**Total**					**991**

The energy consumption of public utility grid is plotted for the 30 weeks is as shown in Fig. 4.

## 4 Experimental Design, Materials and Methods

### 4.1 Solar irradiation of Nile University of Nigeria Abuja

Nile university of Nigeria sits at the heart of Abuja on co-ordinates **9.013898°, 7.396578° (09°00′50″, 007°23′48″).** The campus has a land size of 113 hectares. The solar irradiation data of the university was obtained from the official website of Global Solar Atlas on 30th May 2023. The average hourly profile of the direct normal irradiation is shown in Table 7.

**Table 7:** The average hourly profile of the direct normal irradiation of Nile University of Nigeria.

Average hourly profiles of Direct normal irradiation [Wh/m^2^]													
S/N	Hours	Jan	Feb	Mar	Apr	May	Jun	Jul	Aug	Sep	Oct	Nov	Dec
1	0 - 1	0	0	0	0	0	0	0	0	0	0	0	0
2	1 - 2	0	0	0	0	0	0	0	0	0	0	0	0
3	2 - 3	0	0	0	0	0	0	0	0	0	0	0	0
4	3 - 4	0	0	0	0	0	0	0	0	0	0	0	0
5	4 - 5	0	0	0	0	0	0	0	0	0	0	0	0
6	5 - 6	0	0	0	1	9	10	6	3	6	7	4	0
7	6 - 7	26	21	32	54	81	80	68	66	100	117	182	110
8	7 - 8	187	145	137	143	162	146	109	101	170	236	368	303
9	8 - 9	316	266	242	229	228	196	147	128	216	313	488	433
10	9 - 10	418	365	323	296	282	241	180	151	245	370	573	529
11	10 - 11	493	436	385	353	342	306	239	185	302	441	626	595
12	11 - 12	515	470	415	387	372	312	258	229	352	480	637	609
13	12 - 13	502	459	414	388	380	327	282	241	368	460	614	586
14	13 - 14	442	405	355	345	345	309	275	239	315	402	539	516
15	14 - 15	349	312	272	270	292	271	247	212	270	309	435	416
16	15 - 16	221	199	173	176	214	210	199	176	210	199	295	282
17	16 - 17	78	73	62	66	91	119	125	122	108	48	51	66
18	17 - 18	0	0	0	0	1	16	23	15	3	0	0	0
19	18 - 19	0	0	0	0	0	0	0	0	0	0	0	0
20	19 - 20	0	0	0	0	0	0	0	0	0	0	0	0
21	20 - 21	0	0	0	0	0	0	0	0	0	0	0	0
22	21 - 22	0	0	0	0	0	0	0	0	0	0	0	0
23	22 - 23	0	0	0	0	0	0	0	0	0	0	0	0
24	23 - 24	0	0	0	0	0	0	0	0	0	0	0	0
	Sum	3547	3151	2810	2708	2799	2543	2158	1868	2665	3382	4812	4445

Furthermore, the monthly profile was also obtained and shown in Table 8. With highest DNI of 137.8 KWh/m^2^ In the month of December and 57.9 KWh/m^2^ recorded in August.

The PV Energy potential parameters of Table 10 have significant effect on the installation of the PV solar system. Direct Normal Irradiation which is the amount of solar radiation received per unit area by a surface that is perpendicular to the rays of the sun at its current position in the sky is called Direct Normal Irradiation (DNI) this found to be 1117.3 kWh/m^2^. Similarly, the global horizontal irradiation that shows amount of terrestrial irradiance hitting a surface horizontal to the surface of the earth is found to 1893.9 kWh/m^2^. In the same vein the diffuse horizontal irradiation because of scatter caused by molecules received per unit area by a surface that does not arrive on a direct path from the sun is obtained as 1055 kWh/m^2^.

**Table 8:** Month Average DNI of Nile University Campus.

S/N	Month	Monthly Average Direct Normal Irradiation in kWh/m^2^
1	January	110
2	February	88.2
3	March	87.1
4	April	81.3
5	May	86.8
6	June	76.3
7	July	66.9
8	August	57.9
9	September	80
10	October	104.9
11	November	144.4
12	December	137.8
	Yearly	1121.6

These parameters related to the Nile Universities’ potential for PV energy are shown in Table 9 below.

**Table 9:** PV Energy potential parameters for Nile University Campus.

S/N	Parameter	Abbreviation	Value	Unit
1	Specific photovoltaic power output	PVOUT_specific	1508.9	kWh/kWp
2	Direct normal irradiation	DNI	1117.3	kWh/m^2^
3	Global horizontal irradiation	GHI	1893.9	kWh/m^2^
4	Diffuse horizontal irradiation	DIF	1055	kWh/m^2^
5	Global tilted irradiation at optimum angle	GTI_opta	1930.1	kWh/m^2^
6	Air temperature	TEMP	2 7.2	°C
7	Optimum tilt of PV modules	OPTA	13	°
8	Terrain elevation	ELE	401	m

Additionally, Air temperatures: is the temperature of the ambient air which plays a critical role in PV system performance. However, solar panel temperature usually ranges between 15 °C and 35 °C during which solar cells will produce energy at maximum efficiency [2]. The optimum tilt angle of PV modules is angle required by the solar panels to get their best performance. These other parameters are obtained as 27.2 °C, 13°, and 401 m respectively.

The methodology adopted for each in determining each parameter is shown in Table 10.

**Table 10:** Methodology of PV Energy Potential parameters.

S/N	Acronym	Full name	Unit	Type of Use
1	DIF	Diffuse horizontal irradiation	kWh/m^2^, MJ/m^2^	Average yearly, monthly or daily sum of diffuse horizontal irradiation
2	DNI	Direct normal irradiation	kWh/m^2^, MJ/m^2^	Average yearly, monthly or daily sum of direct normal irradiation
3	ELE	Terrain elevation	m, ft	Elevation of terrain surface above/below sea level
4	GHI	Global horizontal irradiation	kWh/m^2^, MJ/m^2^	Average annual, monthly, or daily sum of global horizontal irradiation
5	GTI	Global tilted irradiation	kWh/m^2^, MJ/m^2^	Average annual, monthly or daily sum of global tilted irradiation
6	GTI opta	Global tilted irradiation at optimum angle	kWh/m^2^, MJ/m^2^	Average annual, monthly or daily sum of global tilted irradiation for PV modules fix-mounted at optimum angle
7	OPTA	Optimum tilt of PV modules	°	Optimum tilt of fix-mounted PV modules facing towards Equator set for maximizing GTI input
8	PVOUT_specific	Specific photovoltaic power output	kWh/kWp	Yearly and monthly average values of photovoltaic electricity (AC) delivered by a PV system and normalized to 1 kWp of installed capacity
9	PVOUT_total	Total photovoltaic power output	kWh, MWh, GWh	Yearly and monthly average values of photovoltaic electricity (AC) delivered by the total installed capacity of a PV system
10	TEMP	A ir temperature	°C,°F	Average yearly, monthly and daily air temperature at 2 m above ground. Calculated from outputs of ERA5 model

## 5 Nile University Power Station Components

In this section, the major component that made up the power station one (PS-1) of Nile University of Nigeria is discussed. From the generators; the diesel engines, the PV solar system, and public utility substation to the measuring instruments; smart meters and the combiner panels are all presented. The Mains supply voltage is usually AC and hence the diesel Generators, the PV solar system and the public utility are used to supply power to a load at “mains” voltage levels in an autonomous way. The general structure of the power station is as shown in Fig. 11. Furthermore, the major source of energy generation systems with their capacities are shown in Table 11.

**Table 11:** PS-1 Energy Generation Sources and their capacity.

S/N	Source of Energy	Manufacture	Quantity	Capacity Kw
1	Diesel Engine 1,2	Marapco	2	400
2	Diesel Engine 3	Caterpillar	1	800
3	Diesel Engine 4	Caterpillar	1	1000
4	PV Solar System	SMA Hybrid Inverters	10	500
5	Public Utility Grid	AEDC	2	1200
Total			6	4300

### 5.1 Diesel generators

The four diesel generating sets in the PS – 1 are used as the primary power source, they operate in a priority format such that; one genset operate all the time to provide power to campus. However, If the load demand increases, one or more generators will be automatically called to start. They will then synchronize onto the bus and provide power in parallel with the other set(s). At this point, the sets connected to the bus will share the load, normally utilizing load sharing equipment. Using multiple generating sets rather than one large set allows for maintenance to be performed on one of the sets while the other set(s) are still available for duty[3].

**Fig. 5:**
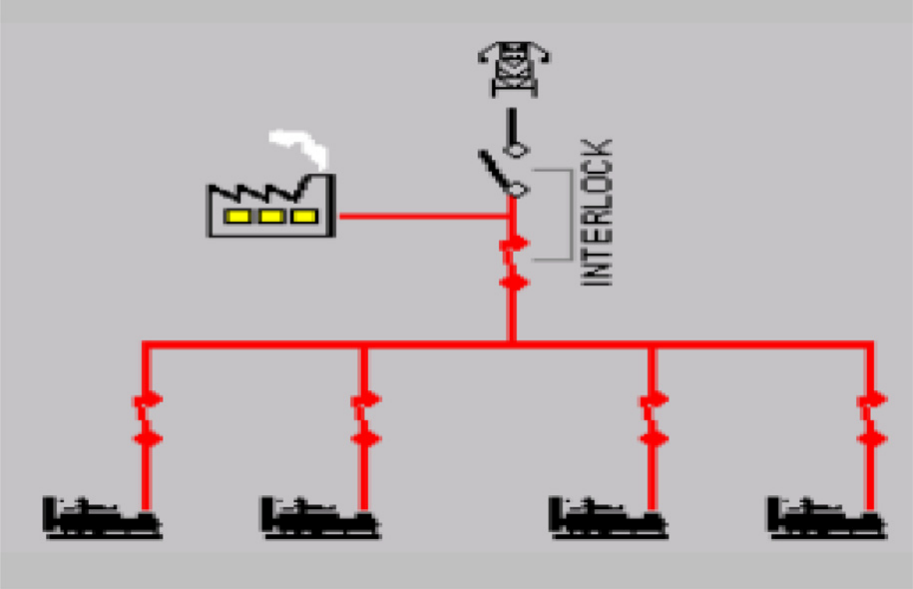
Synchronized diesel generators.

Additionally, if load demands are low, individual generators can be started as required, rather than one large generator being used at (for instance) only 25% of its full load rating. If the overall size of the load increases, for instance due to university expansion another set can be added to increase capacity with minimal disruption to the rest of the system. These generating sets are synchronized in parallel as shown in Fig. 5.

### 5.2 Roof-top grid tied PV solar system

**Fig. 6:**
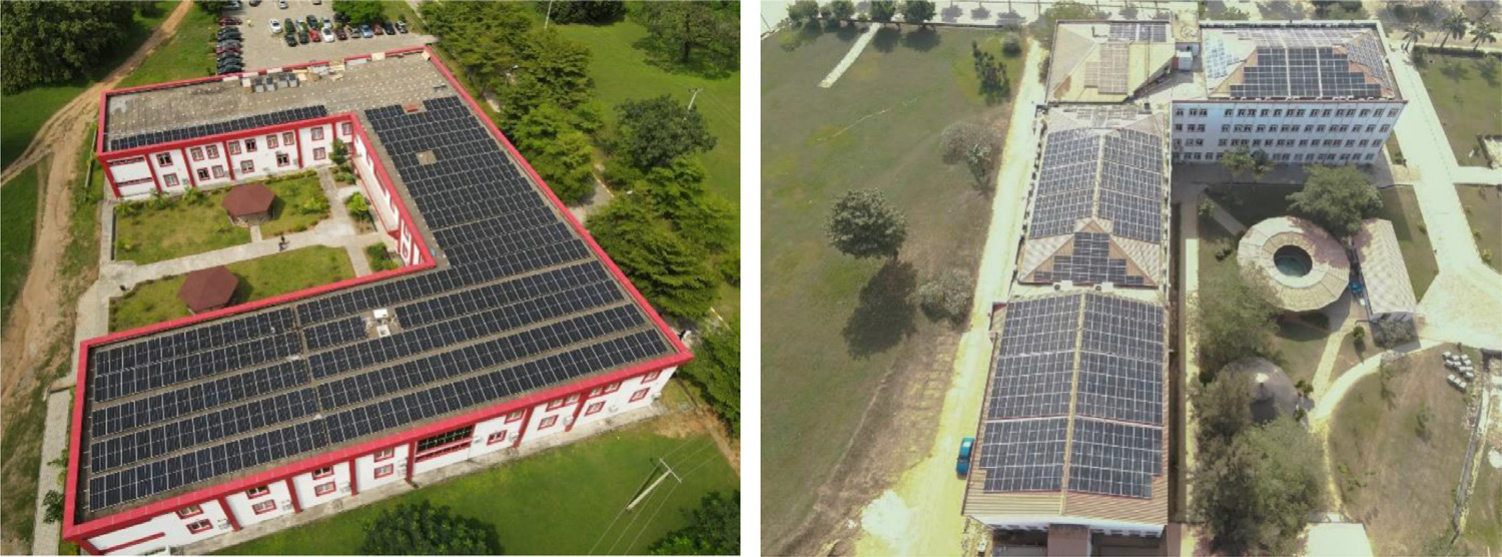
Arial view of the roof-top PV solar system.

The roof-top grid tied PV solar system is as shown in the Fig. 6. This system is installed on lands size area of 2900 sqm with a capacity of 500KVA. It's a grid-tied system that operate only with the availability of energy on the busbar.

### 5.3 PV embodied carbon emissions

A sustainable energy technology can be defined as a technology deployed to power, ventilate, heat and/or cool a building that relies on resources that are environmentally friendly. However, each technology will come with its own associated embodied carbon, which might potentially represent a significant proportion of a building's total embodied carbon impact. Thus, the embodied impact refers to the energy and emissions released to create, manufacture, transport use and dispose each technology.

Furthermore, the impact of some technologies can significantly increase the embodied CO_2_e impact of modern low to zero energy buildings. Considering the whole life CO_2_e impact of each aspect of a building is crucial for the successful creation of a truly low to zero carbon building.

Therefore, the designing of sustainable system relies on the impact of every component in the sustainable energy chain towards the environment. Hence, PV energy system is regarded as one of the most reliable and environment friendly renewable energy technology which has the potential to contribute significantly to a sustainable energy system. It also plays an important role to mitigate C_O_2 emissions. The C_O_2 emissions per year by each component can be calculated as [4].

(3) $$Co2\;emission\;per\;year=\;\frac{Enbodied\;energy\times Average\;Co2\;Intensity}{Lifetime}$$

Energy output of PV system depends on the solar radiation and temperature, etc. Therefore, it is very site specific and variable. Proper sizing and designing of PV system is must for a reliable performance for a long period of time.

### 5.4 Public utility grid

**Fig. 7:**
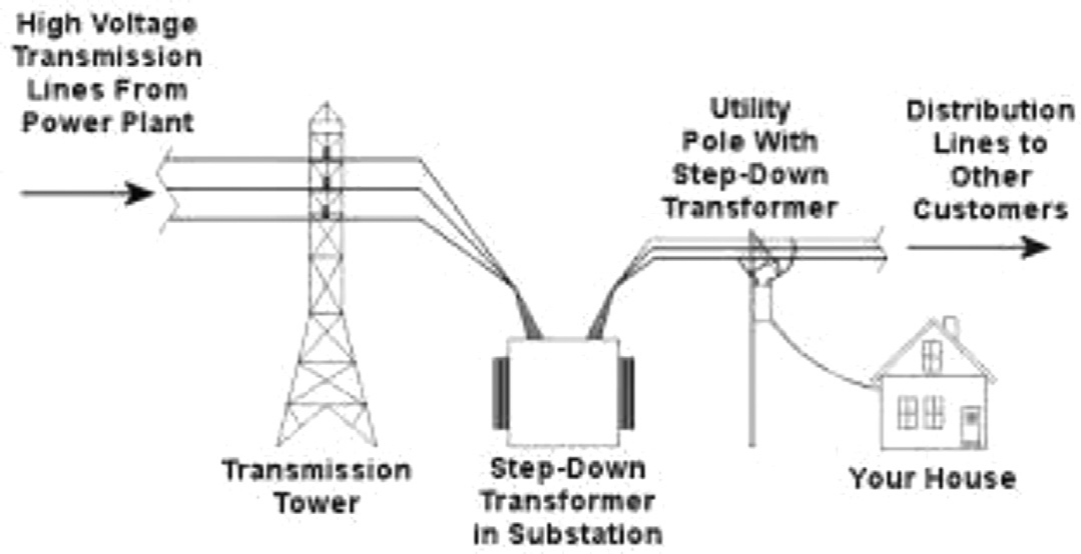
Public utility energy grid.

Abuja Electricity Distribution Company (AEDC) is the main supplier of electricity of the public utility grid a voltage transmission level of 33KV. However, this voltage level is stepped down to 415 V for local consumption. The public utility grid is characterized with high voltage transmission lines from the Nigerian National Grid and two step down transformers as shown in Fig. 7.

## 6 PS-1 Power Management

Together with SMA inverters, the SMA Hybrid Controller is a system solution for the installation of PV power plants and hybrid systems on the basis of gensets or electrical storage systems [5]. The Hybrid Controller continuously monitors the power output of the SMA inverters as well as the operating state of all gensets and loads in the local utility grid. On this basis, the Hybrid Controller controls the SMA inverters and adjusts its output power, where necessary. When supplying micro-grids with energy, the Hybrid Controller additionally takes on different grid management functions as well as the operating reserve provision in large interconnected systems. In combined operation with the genset controllers, the Hybrid Controller must fulfill the following tasks for this:

• Recording Data on the Current Operating State of the Gensets
• Specifying Sufficient Reserve Power of the Gensets

**Fig. 8:**
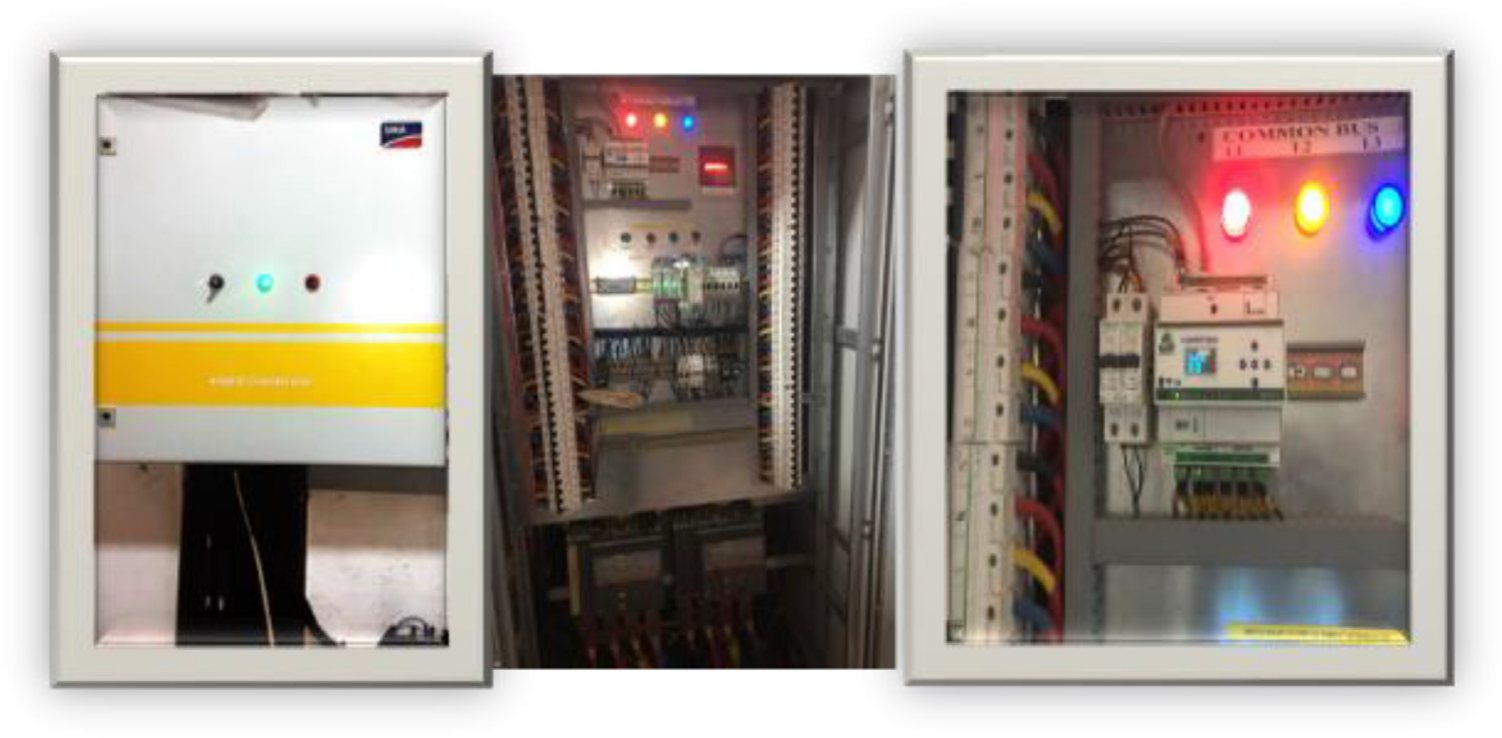
The combiner system.

The combiner panel is the system that combines the output of all the generating sets in single output that feeds the Nile University campus. This is shown in Fig. 8.

**Fig. 9:**
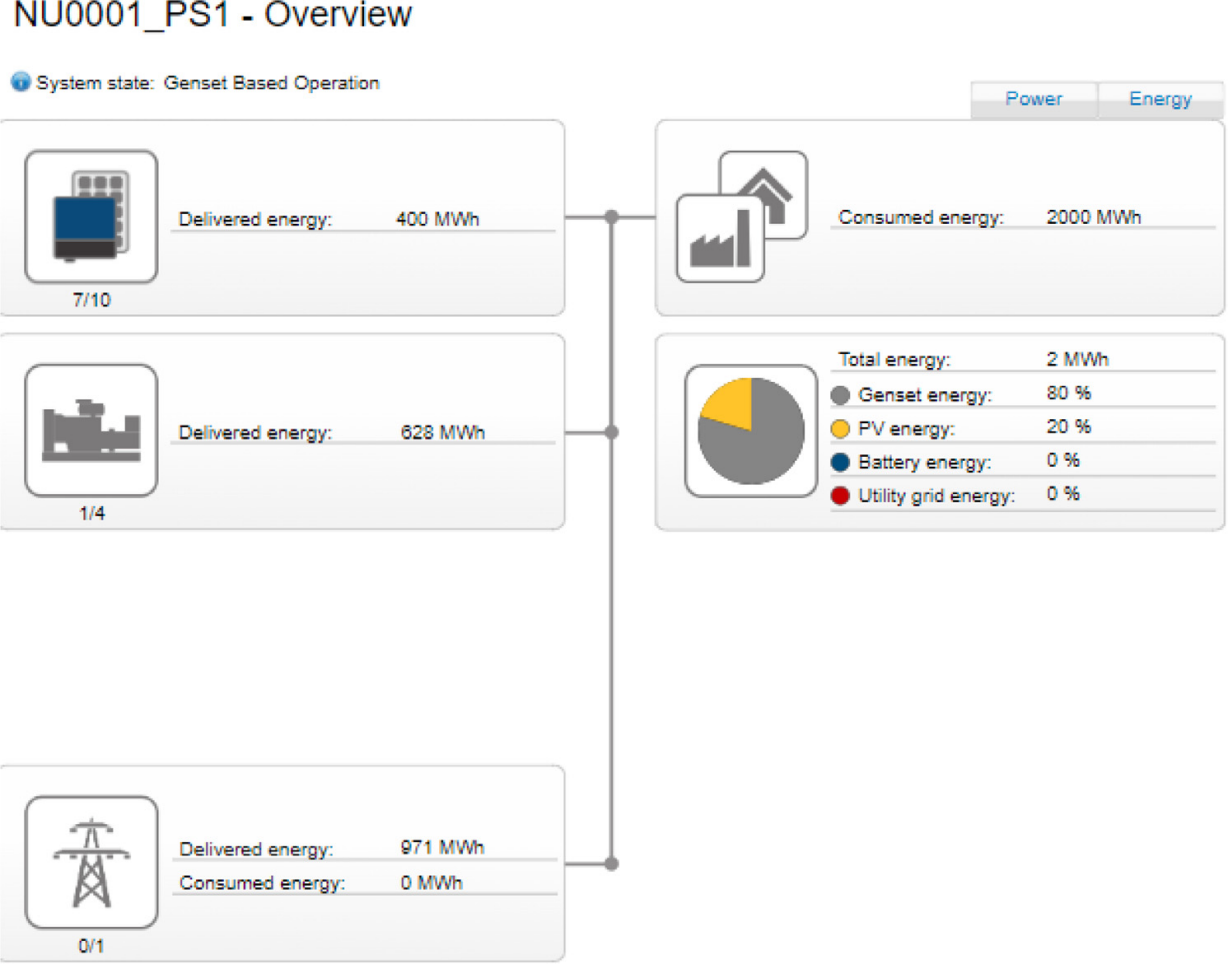
Data measurement for the three sources of energy.

**Fig. 10:**
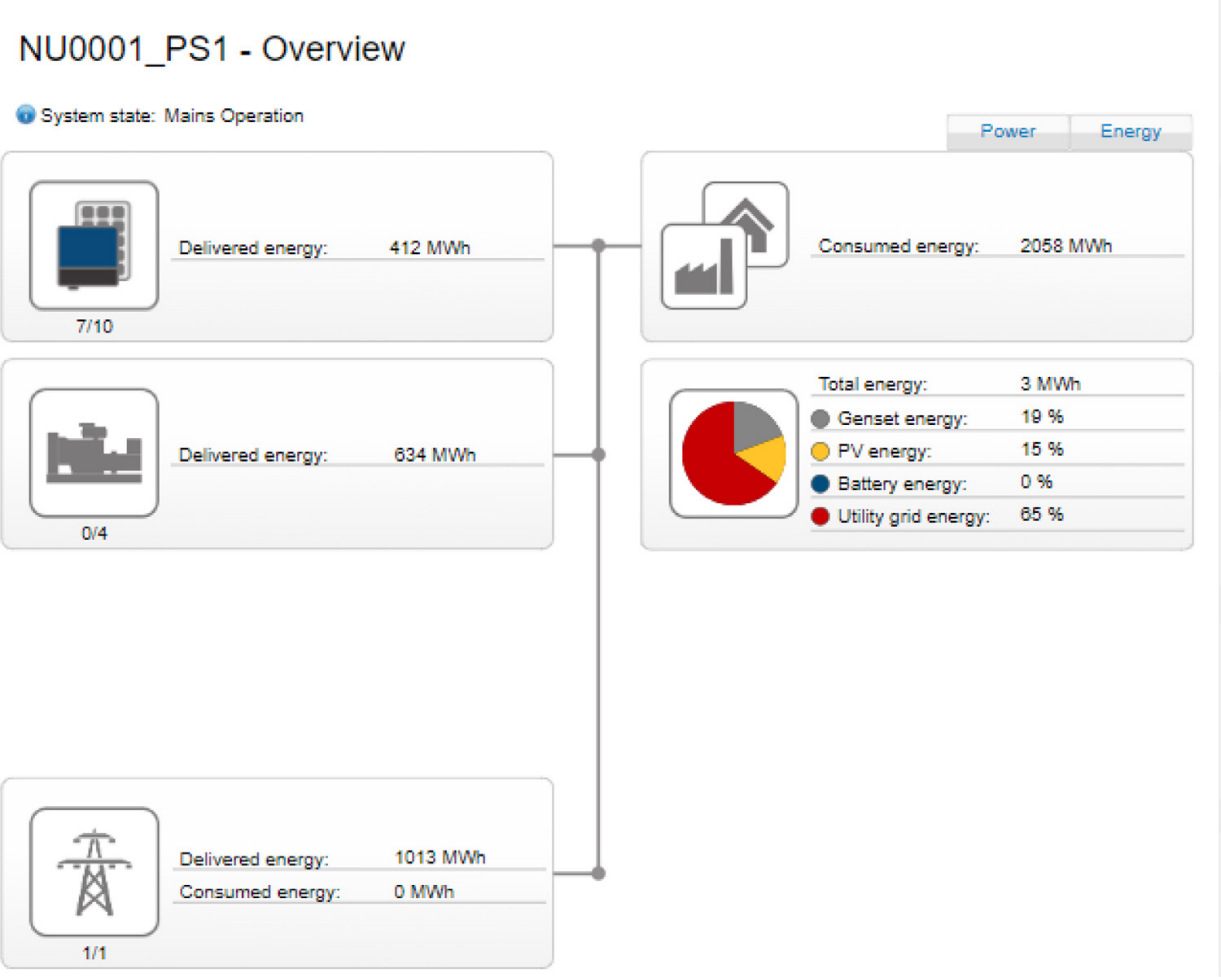
SMA Controller data measurement.

The weekly measurement carried out on each source of power was measurement using an SMA CT which transmit same to the Hybrid controller. The Hybrid controller then display the real-time data readings as shown in the interface shown in Figs. 9-10.

**Fig. 11:**
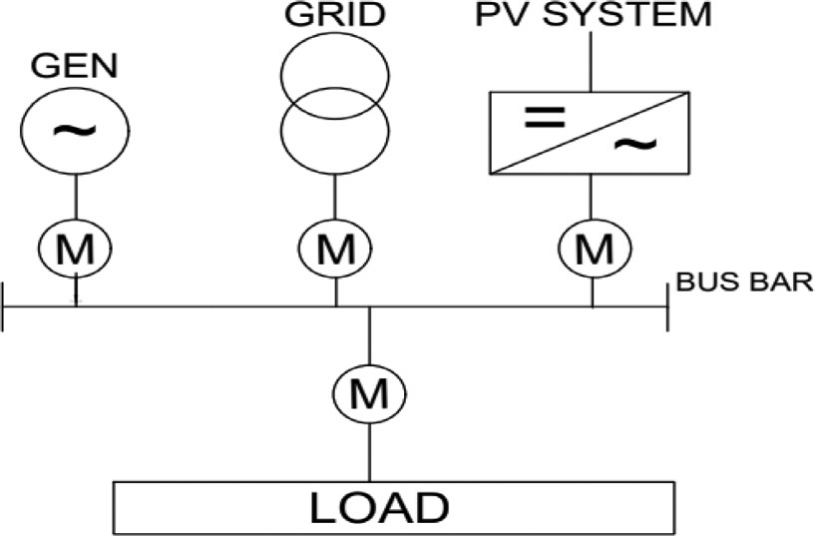
Overall system structure.

As it can be seen, in this figure a total of 2000 MWh of energy was consumed by the various buildings of the university campus. However, 80% of the load was powered using the Gen sets and the remaining 20% was powered by the PV solar system. Furthermore, the utility grid was totally unavailable. Similarly, Fig. 10 shows the scenario where all the three sources have made significant contribution to load demand. In this measurement made by the SMA hybrid controller, it is shown that 2058 MWh was consumed by the load. However, in this particular measurement, only 19% was contributed by gensets, 15% of the load was addressed by the PV solar system and 65% of this load was delivered by the utility grid (Fig. 11).

## 7 The Measurement Instruments

Smart meters are used to measure the output of each generating set. They are made up of current transformers. The key component in smart meters is the current transducer. There is several functional principles for implementation of the current transducer. The shunt resistor is a favorite choice because of the regulations concerning maximum power consumption (2 W per phase acc. to IEC 62053-21, −23), its resistance is limited to some hundreds of µΩ [6] . This low value results in very low secondary voltages at low primary currents. The smart meters are strapped around the coil power lines coming from each the generating sets [7].

In this measurement, the SMA CT used which meets the requirements of the EN 61869-2 standard. They which are designed, among others, to work with energy meters of accuracy class from 0.2 s to 3 s and current ranges from 15 A to 8000 A.

The SMA Energy Meter calculates phase-exact and balanced electrical measured values and communicates these via Ethernet in the local network. In this way, all data on grid feed-in and genset energy as well as PV generation by other PV inverters can be communicated to SMA systems frequently and with a high level of precision. Additionally, the SMA meter used has a measurement accuracy of 1%, and measurement cycle of 1000 ms respectively.

## 8 The Overall System

The overall system is shown in Fig. 9. The figure depicts how the sources of energy are all integrated together at the bus bar of the power station. Furthermore, As seen in the previous sections, the PS1 has Four synchronized Genset with a combined generation capacity of 2.6Mw, a PV solar system with capacity of 500 kW, and a public utility grid with transformer rated 1200MV capacity. These sets are integrated together through the SMA hybrid combiner and control panel which is responsible for the energy management of the source. However, smart meters are used to measure the output of each energy system. These smart meters read the energy data and transmit them via Ethernet to SMA controller. Furthermore, through the SMA hybrid controller interface, data energy consumption data of the campus is obtained.

The PS1 powers Block A, B, C, the female Hostel and staff quarters of university campus.

However, the total energy contribution from each source of energy is as shown in Fig. 10.

## Ethics Statements

Nil.

## CRediT authorship contribution statement

**Tahir A. Zarma:** Conceptualization, Methodology, Software. **Paul O. Micheal:** Data curation, Writing – original draft. **Ahmadu A. Galadima:** Visualization, Investigation. **Tologon Karataev:** Supervision. **Adekunle Adeleke:** Writing – review & editing. **Oghenewvogaga Oghorada:** Writing – review & editing. **Hussein U. Suleiman:** Software, Validation.

## Declaration of Competing Interests

The authors declare that they have no known competing financial interests or personal relationships that could have appeared to influence the work reported in this paper.

## Data Availability

Energy Demand and carbon Emission Data set (Original data) (Mendeley Data).
